# Dynamic chest radiography: a state-of-the-art review

**DOI:** 10.1186/s13244-023-01451-4

**Published:** 2023-06-19

**Authors:** Fred Fyles, Thomas S. FitzMaurice, Ryan E. Robinson, Ram Bedi, Hassan Burhan, Martin J. Walshaw

**Affiliations:** 1 Respiratory Research Group, Liverpool University Hospitals Foundation Trust, Liverpool, UK; 2 Clinical Sciences Department, Liverpool School of Tropical Medicine, Liverpool, UK; 3 Department of Respiratory Medicine, Liverpool Heart and Chest Hospital NHS Trust, Liverpool, UK; 4 Institute of Life Course and Medical Sciences, University of Liverpool, Liverpool, UK; 5 Department of Bioengineering, University of Washington, Seattle, WA USA; 6 Institute of Infection and Global Health, University of Liverpool, Liverpool, UK

**Keywords:** Radiography (Thoracic), X-rays, Thoracic wall, Diaphragm, Lung

## Abstract

Dynamic chest radiography (DCR) is a real-time sequential high-resolution digital X-ray imaging system of the thorax in motion over the respiratory cycle, utilising pulsed image exposure and a larger field of view than fluoroscopy coupled with a low radiation dose, where post-acquisition image processing by computer algorithm automatically characterises the motion of thoracic structures. We conducted a systematic review of the literature and found 29 relevant publications describing its use in humans including the assessment of diaphragm and chest wall motion, measurement of pulmonary ventilation and perfusion, and the assessment of airway narrowing. Work is ongoing in several other areas including assessment of diaphragmatic paralysis. We assess the findings, methodology and limitations of DCR, and we discuss the current and future roles of this promising medical imaging technology.

**Critical relevance statement** Dynamic chest radiography provides a wealth of clinical information, but further research is required to identify its clinical niche.

**Graphical abstract:**

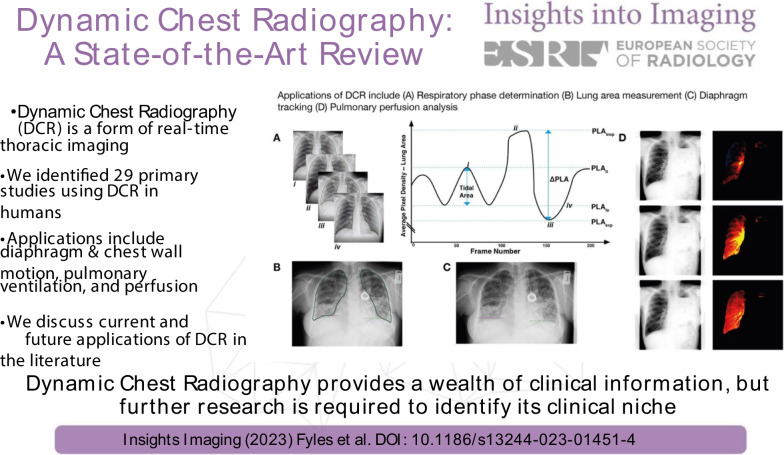

**Supplementary Information:**

The online version contains supplementary material available at 10.1186/s13244-023-01451-4.

## Key points

- Dynamic chest radiography (DCR) captures high-resolution moving images of the thorax.
- The ionising radiation dose of DCR is low.
- DCR can image the diaphragm, chest wall, ventilation and perfusion.
- Most papers on DCR are small, with heterogeneity in study design or outcome.
- Large, multicentre studies with similar outcomes and healthy controls are desirable.

**Supplementary Information:**

The online version contains supplementary material available at 10.1186/s13244-023-01451-4.

## Introduction

**Fig. 1:**
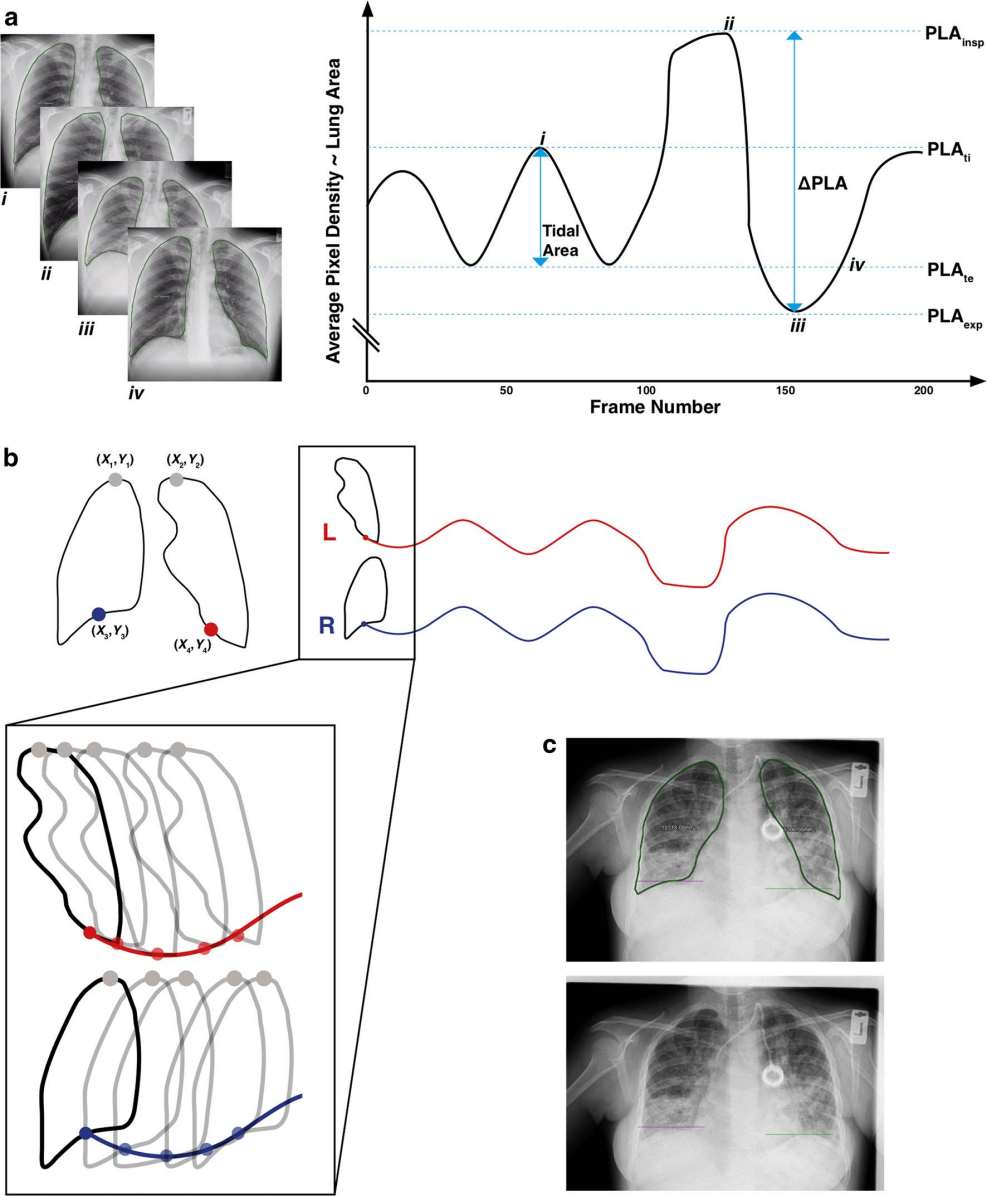
Dynamic chest radiograph interpretation. Visualisation of a dynamic chest radiograph for the purpose of (**a**) identification of phase of breathing via average pixel density change over time and associated projected lung area (PLA) measurements, (**b**) Automated hemidiaphragm midpoint tracking. **c** Shows example images of lung area identification (top) and hemidiaphragm tracking (bottom) in a person with cystic fibrosis bronchiectasis. *PLA* projected lung area, *PLA*_*insp*_ PLA at full inspiration, *PLA*_*exp*_ PLA at maximum expiration, *PLA*_*ti*_ PLA at point of tidal breath in, *PLA*_*te*_ PLA at point of tidal breath out

First described in the early 2000s [1–3], dynamic chest radiography (DCR) is a real-time X-ray imaging system that takes sequential images of the thorax in motion usually over 10 to 20 s. A high-resolution flat panel detector (FPD) produces temporal and spatial digital images with a wide field of view (FOV) that captures the entire thorax. The temporal resolution of DCR can be set as high as 15 frames per second (fps), above the minimum necessary to capture rapid movement. Recent hardware advances and segmentation-based proprietary image processing software automatically identify moving structures, such as the diaphragm and visible posteroanterior (PA) lung area, or to assess the change in pixel intensity of lung tissue over the breathing cycle [4]. When segmentation of lung parenchyma and vasculature is applied, changes in pixel density can infer ventilation and perfusion respectively without the need for intravenous contrast agents or inhaled tracers [5]. Depending on exposure settings, a single DCR image can be adequate for diagnostic purposes and is visually similar to a standard (‘plain’) PA chest radiograph. A visual description of a DCR image series and its interpretation is shown in Fig. 1, and a technical description of the image processing methodology can be found as a Additional file 1.

**Fig. 2:**
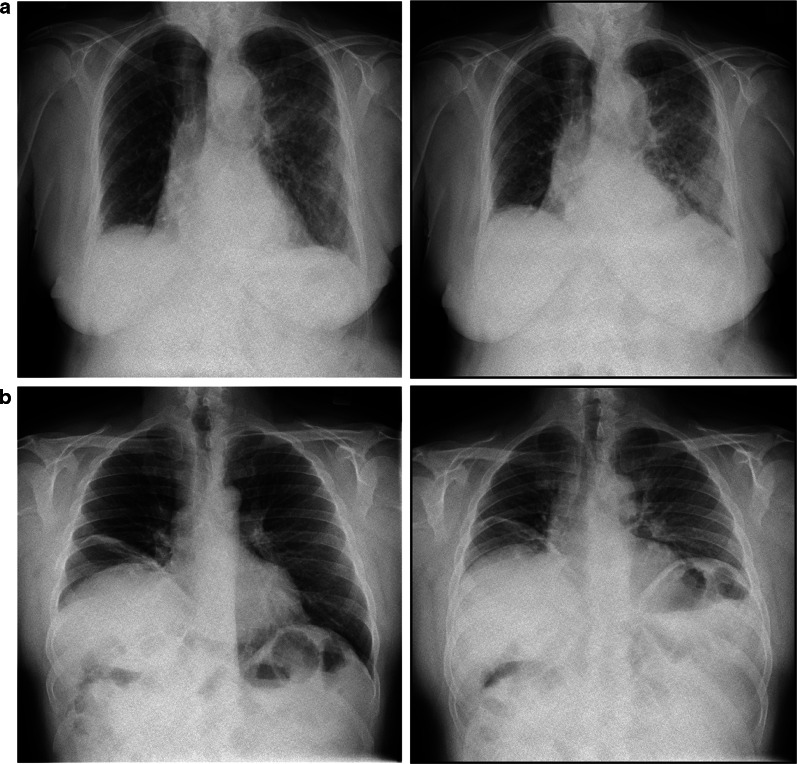
Demonstration of use of dynamic chest radiography in lung parenchymal and diaphragmatic disorders. Still frame at full inspiration shown on left, and still frame at expiration on the right. **a** DCR post-left lower lobectomy complicated by pneumonia, in a female in her 70 s, demonstrating left mid- and lower-zone consolidation, with normal diaphragmatic movement. **b** DCR in a male in his 50 s, demonstrating an elevated right hemidiaphragm; paradoxical right diaphragm movement on sniffing consistent with phrenic nerve palsy

**Fig. 3:**
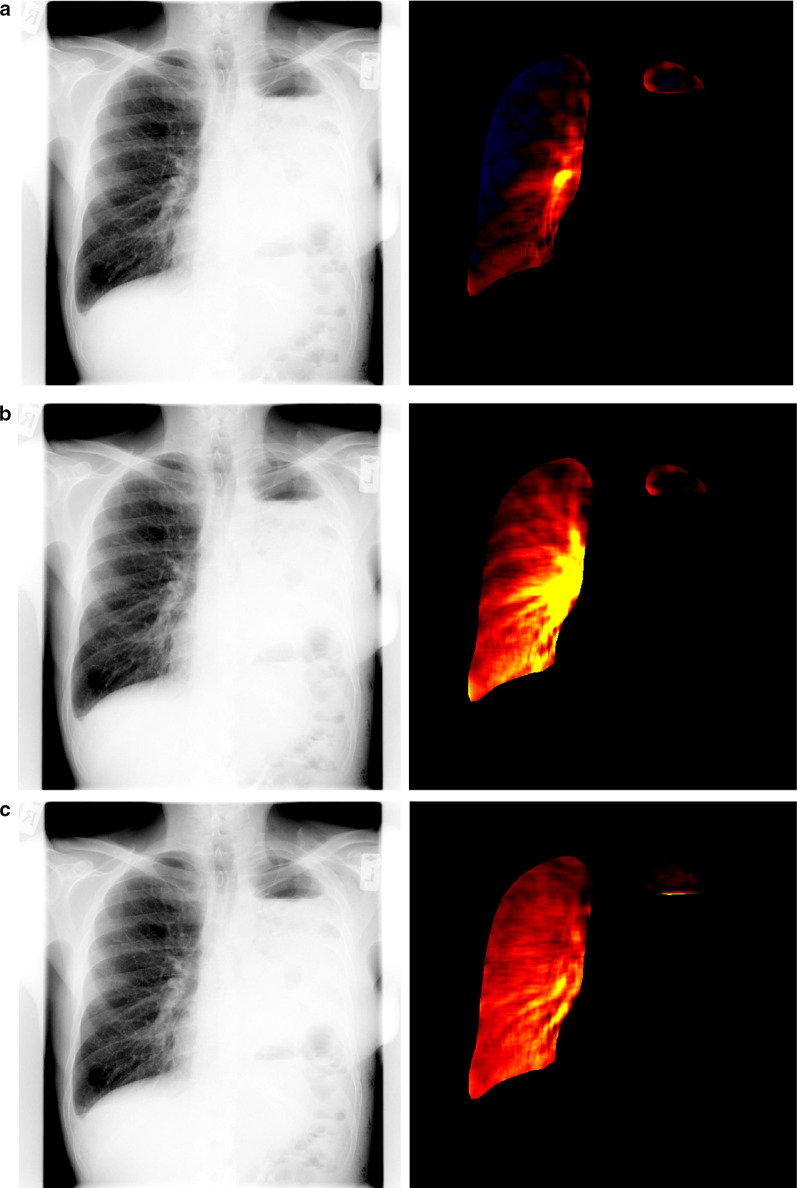
Demonstration of use of dynamic chest radiography to detect pulmonary blood flow. DCR in a male in his 50 s following left pneumonectomy. Standard DCR images is shown in lef-thand panels, DCR perfusion mapping in right. Still images prior to (**a**), during (**b**) and after (**c**) right ventricular contraction

DCR has several advantages as an imaging technology: the equipment has an identical footprint to a standard chest radiography unit and image acquisition confers a lower radiation dose than CT or traditional fluoroscopy. DCR is typically performed in an upright position (although it may be carried out supine), without the necessity for the forced manoeuvres or use of mouthpieces that are required during air flow-based lung function measurement techniques such as spirometry or plethysmography, which interfere with normal respiratory or cardiovascular physiology [6, 7]. The image capture process is quick, taking little more time than a standard chest radiograph [8]. A full list of the exposure settings used by our group can be found as a Additional file 1. Examples of DCR applications are shown in Figs. 2 and 3. A variety of potentially useful clinical information can be derived from a single DCR image series, including diaphragm motion, chest wall motion, large airways diameter and ventilation/perfusion. Information on these parameters is important in a variety of different health conditions including diaphragm palsy, restrictive and obstructive lung diseases, and pulmonary vascular disease. For this reason, DCR may be a technology of interest for research into various respiratory conditions or symptoms.

Since the first description of DCR a steady stream of papers has entered the literature, however to our knowledge no systematic review of the clinical applications and findings of DCR has been carried out. Our objective was to summarise existing DCR knowledge, produce a narrative description of its current application in medical practice, and explore directions for future research.

## Materials and methods

A literature review was conducted using the PRISMA statement for systematic reviews to guide the process; the PRISMA checklist can be found as an Additional file 1. The study is registered in the PROSPERO database, registration number CRD42022328181.

### Search strategy

A comprehensive MEDLINE (1990–January 2022) and EMBASE (1990–January 2022) protocol-driven search was performed. Search terms included ‘dynamic chest x-ray’, ‘DCR’ and ‘dynamic thoracic imaging’. The full list is detailed as a Additional file 1. References from relevant publications and abstracts from major respiratory conferences were reviewed, and clinical trial registers searched. Any additional publications identified during the hand-search were included if they met all inclusion criteria below.

### Study inclusion, data extraction and quality assessment

The following inclusion criteria were used: (1) applies to dynamic chest imaging, (2) English language, (3) performed in humans. Publications were excluded if no full text could be retrieved (e.g. inaccessible despite use of interlibrary loan services). Case reports and narrative reviews were excluded. As the scope of this review was a narrative description of the state of the art, quality was not used as an exclusion criterion. The full texts of studies meeting the inclusion criteria based on their titles and abstracts were obtained and screened by two independent reviewers with several years’ experience of DCR (TSF, 5 years’ experience and RER, 5 years’ experience). A third author (FF, 4 years’ experience) reviewed the list and adjudicated discussions about study inclusion. Data extraction was performed by three independent reviewers (RER, TSF, FF). The study design, sample population, study objectives, statistical analysis and outcomes measured were collated and presented in a table. The Newcastle–Ottawa scale was applied for non-randomised studies and the Joanna Briggs Institute (JBI) Checklist for cross-sectional studies, in order to guide inclusion or exclusion. None of the studies identified were randomised. A narrative synthesis was then created from these papers.

## Results

The initial search identified 826 papers published January 2006 to January 2022; 791 papers were excluded as they were either duplicates, or not relevant to the aim of the review after the titles and abstracts had been screened for eligibility. A further 72 articles were identified by a combination of citation searches and website searches. A total of 106 full-text articles were then assessed, 29 of which met the inclusion criteria (see Table 1).

**Table 1:** Summary of all included studies

Author	Study design	Focus	Subjects	N	Study objectives	JBI quality score*
Tanaka et al. [8]	Case series	Pixel value analysis	Healthy volunteer(s), COPD	18	Assess correlation between diaphragm motion parameters and lung vital capacity. Describe methods for visualising change in pixel value and compare to clinical/radiological data	5
Tanaka et al. [4]	Observational	Technical report	NA	37	Compare four different automatic processes with accuracy of manual selection by radiologist to determine max inspiration/expiration	2
Tanaka et al. [9]	Observational	Ventilation	Healthy volunteer(s)	6	Assess average pixel value change during respiratory cycle and regional differences in pixel value change in standing and decubitus position	6
Tanaka et al. [10]	Observational	Ventilation, perfusion	Healthy volunteer(s)	7	Assess feasibility of using DCR to map blood distribution for future clinical use	3
Kawashima et al. [11]	Observational	Reproducibility	Healthy volunteer(s)	5	Assess reproducibility of changes in pixel value between repeated DCR	2
Tanaka et al. [12]	Observational	Perfusion	Various	14	To compare quantitative pulmonary blood flow using DCR and perfusion scanning	5
Tanaka et al. [5]	Case control	Perfusion	Various	20	To assess the validity of DCR for evaluating pulmonary blood flow distribution, with normal controls	3
Tsuchiya et al. [13]	Observational	Nodule motion analysis	Healthy volunteer(s)	8	To detect lung nodules (simulated)	4
Tanaka et al. [14]	Case control	Ventilation	Various	20	To assess the ability of DCR to detect ventilatory impairment using pixel value change, compared with scintigraphy	5
Tanaka et al. [15]	Case control	Rib motion	Various	16	To assess the ability of DCR to detect rib motion in normal controls and individuals with scoliosis	3
Yamada et al. [16]	Observational	Diaphragm motion	Healthy volunteer(s)	172	To evaluate the average diaphragmatic excursions in healthy volunteers, and assess the relationships between DCR metrics and anthropometrics/spirometry	5
Tanaka et al. [17]	Observational	Ventilation	Various	30	To assess ventilatory defects using change in lung texture	4
Yamada et al. [18]	Case control	Diaphragm motion	Healthy volunteer(s), COPD	86	Evaluate the difference in tidal breathing diaphragm motion between COPD and healthy controls using DCR	5
Yamada et al. [19]	Case control	Craniocaudal gradient analysis	Healthy volunteer(s), COPD	90	Evaluate the difference in craniocaudal gradient of maximum pixel value change rate between COPD and healthy controls	5
Hida et al. [20]	Observational	Diaphragm motion	Healthy volunteer(s)	174	To assess diaphragm motion in standing positions during forced breathing, and evaluate its associations with demographics and pulmonary function tests	7
Hida et al. [21]	Case control	Diaphragm motion	COPD, healthy	62	To assess differences in diaphragmatic motion (speed and excursion) between COPD and control. To assess correlation between pulmonary function tests and diaphragmatic motion	7
Kitahara et al. [22]	Observational	Segmentation	Various	214	To develop a lung segmentation for dynamic chest radiography, and to assess the clinical utility of this measure for pulmonary function assessment	3
Hanaoka et al. [23]	Diagnostic cohort study	Pulmonary function	Lung cancer resection	52	To assess the use of DCR to calculate post-operative pulmonary function compared to pulmonary perfusion scintigraphy	7
Hino et al. [24]	Observational	Lung areas	Healthy volunteer(s)	162	To investigate correlation of projected lung areas with pulmonary function	7
Ohkura et al. [25]	Observational	Ventilation	COPD	118	Assess relationship between lung area (max and min) and rate of change with pulmonary function tests	5
Tanaka et al. [26]	Case control	Ventilation, perfusion	Various	53	To assess the ability of DCR to detect ventilatory impairment using pixel value change, compared with ventilation/perfusion imaging	6
Watase et al. [27]	Case control	Tracheal diameter analysis	COPD	40	To assess the ability of DCR to detect intrathoracic tracheal narrowing between normal and abnormal cases	4
Yamamoto et al. [28]	Observational	Perfusion	Various	42	Assess the success rate of deep-breath-holding and breath-holding DCR in assessment of pulmonary perfusion; correlation between diaphragm motion and anthropometrics	6
FitzMaurice et al. [29]	Observational	Diaphragm motion, lung areas	Cystic fibrosis bronchiectasis	24	To describe changes in diaphragm motion and lung areas before and after modulator therapy in adults with cystic fibrosis bronchiectasis using DCR	7
FitzMaurice et al. [30]	Case series	Diaphragm motion	Diaphragm palsy	21	To describe diaphragm motion in individuals with a paralysed hemidiaphragm using DCR	6
Ohkura et al. [31]	Case control	Diaphragm motion, lung areas, tracheal diameter	COPD, restrictive lung disease	273	Identify relationship between lung disease (restrictive and obstructive) and parameters on DCR	4
Tanaka et al. [32]	Observational	Ventilation, perfusion	Lung cancer	42	To assess the ability of DCR to detect ventilatory impairment using pixel value change, compared with ventilation/perfusion imaging	5
Ueyama et al. [33]	Case control	Lung volume measurement	Interstitial lung disease	97	To evaluate the ability of DCR to predict forced vital capacity	7
FitzMaurice et al. [34]	Observational	Diaphragm motion, lung areas	Cystic fibrosis bronchiectasis	20	To describe diaphragm motion in individuals undergoing treatment for a pulmonary exacerbation of cystic fibrosis bronchiectasis	7

*DCR* dynamic chest radiography, *COPD* chronic obstructive pulmonary disease

*Point-by-point score is listed in the Additional file 1

### Exposure settings and ionising radiation dose

Device exposure settings and estimated ionising radiation dose are described in Table 2. Image acquisition time ranged from 4 to 37 s, with a median of 10 s; 3 studies did not describe imaging time. Frame rate ranged from 3 to 30 fps, with a median and mode of 15 fps. Earlier studies tended to use a lower frame rate (as low as 3 fps), with later studies almost all using 15 fps. Ionising radiation dose of DCR was not recorded by 3 studies. In those studies where it was reported, the median entrance surface dose (ESD, the measure of radiation absorbed by the skin) was 0.65 mGy, with the highest reported dose being 1.5 mGy. For lateral DCR image series, a dose of 4 mGy was reported. The dose in the lateral view is unsurprisingly higher, due to the need for the X-ray penetration of a greater depth of tissue than in the PA projection. In 3 studies, the radiation exposure was reported in terms of effective dose (ED) and ranged from 0.2 to 0.25 mSv. For comparison, the average ED for a plain PA chest radiograph in the UK is 0.014 mSv [35], an ultra-low-dose CT 0.08 mSv [36] and a high-resolution helical computed tomography (CT) chest scan typically around 9.7 mSv [37]. The imaging protocol varied significantly between studies. However, most studies acquired erect images, with tidal and/or deep breathing manoeuvres overseen by a clinician for diaphragm/chest wall assessment, and constant inspiratory breath holding for pulmonary perfusion analysis [5]. Instructions were spoken [18, 29] or pre-recorded [33].

**Table 2:** Exposure settings and radiation dose of included studies

Author	Duration (s)	ESD (mGy)	Frame rate (fps)
Tanaka et al. [8]	10	0.4	3
Tanaka et al. [4]	10	0.4	3
Tanaka et al. [9]	10	0.4	3
Tanaka et al. [10]	4	0.32	6
Kawashima et al. [11]	8	*NA*	7.5
Tanaka et al. [12]	4	0.6	7.5
Tanaka et al. [5]	4	0.6	7.5
Tsuchiya et al. [13]	10	0.4	3
Tanaka et al. [14]	8	0.6	7.5
Tanaka et al. [15]	10	0.4	3
Yamada et al. [16]	10.0–15.0	0.3–0.5	15
Tanaka et al. [17]	14	*NA*	15
Yamada et al. [18]	10.0–15.0	0.3–1	7.5–30
Yamada et al. [19]	10.0–15.0	0.3–1	7.5
Hida et al. [20]	17–37	0.3–1	15
Hida et al. [21]	10.0–15.0	0.3–1	7.5–15
Kitahara et al. [22]	10	*NA*	15
Hanaoka et al. [23]	10	1.5	15
Hino et al. [24]	10.0–15.0	0.3–1	15
Ohkura et al. [25]	14	EED: 0.21 mSv	15
Tanaka et al. 2020 [26]	*NA*	< 1.9	15
Watase et al. 2020 [27]	15	1.3	15
Yamamoto et al. 2020 [28]	6.0–14.0	1–1.8	15
FitzMaurice et al. 2022 [29]	10	EED: 0.13 mSv	15
FitzMaurice et al. 2022 [30]	20	EED: 0.25 mSv	15
Ohkura et al. 2021 [31]	14	EED: 0.2 mSv	15
Tanaka et al. 2021 [32]	14	< 1.9	15
Ueyama et al. 2021 [33]	18.3–19.6	1.5–4	15
FitzMaurice et al. 2022 [34]	10	EED: 0.13 mSv	15

*ESD* entrance surface dose [of radiation], *EED* estimated effective dose[of radiation]

### Diaphragm and thoracic structure motion

Several studies have used DCR to assess diaphragm motion in healthy volunteers [16, 20] and in respiratory conditions including COPD [21] and cystic fibrosis [29, 34] (see Table 3). DCR-recorded diaphragm motion over the course of a forced breathing cycle appears similar to a spirogram (see Fig. 1), and this motion, from which speed and excursion can be calculated, has been used standalone or to derive further clinical parameters [4].

**Table 3:** Diaphragm excursion and peak inspiratory speed measured using DCR

Paper	N	Population	Age (years)	Height (cm)	Hemidiaphragm excursion*				Hemidiaphragm speed (peak)*			
					R deep	L deep	R tidal	L tidal	R deep	L deep	R tidal	L tidal
FitzMaurice et al. [30]	21	Contralateral (un-paralysed) side of diaphragm palsy cases	61 ± 13	172 ± 10	25 ± 6	33 ± 15	NA	NA	40 ± 16	55 ± 21	NA	NA
FitzMaurice et al. [29]	24	Cystic fibrosis (before modulator drug treatment)	27 ± 6	167 ± 10	18 ± 11	21 ± 11	11 ± 3	14 ± 6	22 ± 14	28 ± 11	13 ± 4	15 ± 4
		Cystic fibrosis (after modulator drug treatment)	27 ± 6	167 ± 10	26 ± 9	31 ± 11	14 ± 6	16 ± 8	31 ± 11	37 ± 16	16 ± 5	18 ± 6
FitzMaurice et al. [34]	20	Cystic fibrosis (during a pulmonary exacerbation)	25 ± 7	164 ± 9	13 (6)	18 (11)	11 (7)	12 (6)	10 (6)	13 (12)	9 (6)	9 (4)
		Cystic fibrosis (after treatment)	25 ± 7	164 ± 9	19 (14)	25 (16)	13 (10)	15 (10)	15 (12)	18 (11)	10 (4)	12 (5)
Hida et al. [20]	174	Healthy volunteers	57 ± 10	163 ± 9	49 ± 17	52 ± 16	NA	NA	27 ± 10	32 ± 12	NA	NA
Hida et al. [21]	15	COPD–GOLD 1–2	74 ± 10	163 ± 5	54 ± 17	61 ± 14	NA	NA	21 ± 9	25 ± 8	NA	NA
	16	COPD–GOLD 3–4	70 ± 9	163 ± 6	40 ± 15	44 ± 14	NA	NA	23 ± 10	26 ± 7	NA	NA
	31	Healthy volunteers	64 ± 5	162 ± 9	53 ± 15	40 ± 13	NA	NA	25 ± 10	34 ± 14	NA	NA
Yamada et al. [18]	47	Healthy volunteers	55 ± 10	162 ± 9	NA	NA	10 ± 4	15 ± 4	NA	NA	12 ± 4	17 ± 4
	39	COPD	71 ± 8	163 ± 6	NA	NA	15 ± 6	17 ± 5	NA	NA	16 ± 5	19 ± 5
Yamada et al. [16]	172	Healthy volunteers	56 ± 10	164 ± 9	NA	NA	11 ± 4	15 ± 5	NA	NA	12 ± 4	16 ± 4

*Expressed as mean ± standard deviation, or median and interquartile range (IQR)

Some studies have explored the role of DCR to quantify motion of the diaphragm as a marker of pulmonary function, exploring its relationship to traditional lung function tests such as spirometry [16, 20, 21]. In healthy individuals, average (mean ± standard deviation) diaphragm excursion during forced deep breathing was found to be 49 ± 17 mm on the right-hand side and 52 ± 16 mm on the left [20]. During tidal breathing the excursion was found to be smaller, at 11 ± 4 mm on the right-hand side and 15 ± 5 mm on the left [16]. The range of hemidiaphragm excursion observed using DCR is similar to that observed using M-mode ultrasound [38]. Clear differences in diaphragm motion have been observed using DCR between healthy volunteers and those with COPD [18], between differing severities of COPD [21] and before and after treatment of pulmonary exacerbations of cystic fibrosis bronchiectasis [34]. DCR-measured diaphragm position at rest has been used as a marker of air trapping [29], with a higher resting diaphragm position associated with improvement in respiratory function after cystic fibrosis transmembrane conductance regulator (CFTR) modulator treatment for adults with cystic fibrosis bronchiectasis [29]. In individuals with COPD, deep breathing diaphragm excursion has been demonstrated to be significantly less than that in healthy individuals [21].

The real-time visual properties of DCR have been used to diagnose diaphragm dysfunction [30], as a form of low-dose fluoroscopy, in which paradoxical diaphragm motion can not only be observed but also quantified by computer assisted tracking of the diaphragm midpoint. Paradoxical diaphragm motion was clearly identified and agreed with the findings of either standard fluoroscopy or ultrasound. Work is ongoing to further explore the use of DCR in cases of suspected diaphragm palsy, with comparison to other reference techniques [39]. To our knowledge, only two studies have compared healthy individuals to those with respiratory disease [16, 20], although no detailed case matching was undertaken. One study tracked rib motion during respiratory manoeuvres [15]. To our knowledge, this has not been applied to a large number of normal individuals, or in the identification of specific pathology with reference to other thoracic cage motion imaging techniques such as structured light plethysmography [40]. DCR has also been applied to lung nodule motion analysis in a single small case series [13].

### Lung area change

Lung area, the visible area of lung tissue visible in either the PA or lateral plane, is variously described as ‘projected lung area’ (PLA) or ‘lung field area’ [24, 25, 29, 34]. An example of a calculated lung field area is shown in Fig. 1. Inspiratory PLA (PLA_insp_) has been found to correlate well with forced vital capacity (FVC) in healthy individuals [24] and those with respiratory disease [29, 31, 33, 34]. These findings are detailed in Table 4. This mirrors well-established associations between FVC and PLA measured on traditional, static chest radiographs [41]. Further correlations between DCR-derived parameters and pulmonary function test (PFT) results have been explored, highlighting the role DCR may plan as an adjunct in patients whose physiological baseline precludes traditional spirometric testing. The relationship between PLA and FEV_1_ is variable. PLA_insp_ demonstrates correlation with FEV_1_ in both healthy individuals[24] and those with restrictive lung diseases [31, 33]. However, this relationship was not demonstrated among patients with COPD, where FEV_1_ is a key marker of disease state [31]. Similarly, whilst change in PLA (ΔPLA) showed moderate correlation with FEV_1_ within a group of individuals with cystic fibrosis bronchiectasis [34], there was only a weak correlation between in those with COPD [25]. These findings may be related to underlying pathophysiology (e.g. presence of emphysema or air trapping) or the low number of COPD patients enrolled in said studies. In restrictive disease, PLA_insp_ has been shown to be significantly reduced [31]. Similarly, patients with severe obstructive lung disease have significantly increased PLA_insp_ and reduced ΔPLA compared to healthy controls, likely reflecting underlying air trapping [25, 31]. DCR has only been used as a marker of response to treatment in individuals with cystic fibrosis bronchiectasis [29, 34]. In these studies, reduced expiratory PLA (PLA_exp_) following treatment for pulmonary exacerbations was postulated to reflect reduced air trapping.

**Table 4:** Posteroanterior projected lung areas as measured using dynamic chest radiography

Paper	N	Population	Age (years)	Height (cm)	PLA_insp_ (PA)* (cm^2^)		PLA_exp_ (PA)* (cm^2^)		Change in PLA (ΔPLA) (cm^2^)	Correlation between PLA_insp_ and FVC
					R	L	R	L		
FitzMaurice et al. [30]	21	Contralateral (un-paralysed) side of diaphragm palsy cases	61 ± 13	172 ± 10	N/A	N/A	N/A	N/A	N/A	N/A
FitzMaurice et al. [29]	24	Cystic fibrosis (pre modulator drug treatment)	27 ± 6	167 ± 10	417 ± 81		334 ± 712		83 ± 40	0.69 (*p* < 0.001)
		Cystic fibrosis (post modulator drug treatment)	28 ± 6	167 ± 10	416 ± 75		299 ± 72		117 ± 37	0.55 (*p* < 0.005)
FitzMaurice et al. [34]	20	Cystic fibrosis (during pulmonary exacerbation)	25 ± 7	164 ± 9	402 ± 67		321 ± 57		29 ± 14	0.69 (*p* < 0.001)
		Cystic fibrosis (after treatment)	25 ± 7	164 ± 9	392 ± 61		303 ± 52		35 ± 10	N/A
Hino et al. [24]	162	Healthy volunteers	57 ± 10	163.4 ± 9	241 ± 30	201 ± 32	173 ± 30	141 ± 27	N/A	0.68 (*p* < 0.001)
Okhura et al. [25]	118	Airflow Limitation	71 ± 8	N/A	N/A	N/A	N/A	N/A	N/A	0.67 (*p* < 0.05)
Ohkura et al. [31]	273	All participants	N/A	N/A	N/A	N/A	N/A	N/A	N/A	0.5 (*p* < 0.05)
	104	Normal spirometry	67 ± 10	162 ± 9	N/A	N/A	N/A	N/A	N/A	N/A
	108	Mild airflow limitation (FEV_1_ > 80% predicted)	72 ± 7	162 ± 9	N/A	N/A	N/A	N/A	N/A	N/A
	25	Moderate airflow limitation (FEV_1_ 50–80% predicted)	71 ± 4	163 ± 7	N/A	N/A	N/A	N/A	N/A	N/A
	6	Severe airflow limitation (FEV_1_ < 50% predicted)	64 ± 16	167 ± 8	N/A	N/A	N/A	N/A	N/A	N/A
	14	Moderate restrictive disease (FVC 65–80% predicted)	70 ± 10	160 ± 10	N/A	N/A	N/A	N/A	N/A	N/A
	16	Severe restrictive disease (FVC < 65% predicted)	60 ± 14	163 ± 8	N/A	N/A	N/A	N/A	N/A	N/A
Ueyama et al. [33]	97	Interstitial lung disease	73 (IQR: 66–77)	162 (IQR: 156–166)	N/A	N/A	N/A	N/A	N/A	0.73 (*p* < 0.001)

*Reported as combined right and left area if reported so in the source paper; *IQR* interquartile range, *FEV*_*1*_ forced expiratory volume of air in 1 s, *FVC* forced vital capacity, *PA* posteroanterior, *PLA* projected lung area

### Ventilation and perfusion imaging

DCR has been proposed as a non-contrast imaging modality to assess ventilation and perfusion (V/Q), potentially addressing the time-consuming and expensive limitations of traditional nuclear medicine V/Q scanning, or the high ionising radiation doses and contrast agent exposure of CT angiography [8]. Preliminary studies have established that DCR can detect ventilation as a change in pixel density and related signal characteristics over time, and can be applied to detect regional differences in ventilation [4, 8, 12, 14, 42].

Few large-scale studies of DCR-derived measures of ventilation have been carried out [14, 17, 19, 32]. Cranio-caudal gradient of mean pixel contrast change rate (the average maximum rate of pixel contrast change in relation to distance from lung apex) has been studied as a marker of obstructive lung disease. This gradient has been shown to be significantly reduced in those with COPD compared to controls, and those with severe (GOLD COPD severity score 3–4) compared to mild (GOLD 1–2) COPD [19]. This finding may be explained by obstruction more markedly affecting lower airways and suggests that DCR may be used in evaluation of severe obstruction, particularly where spirometry may not be possible. However, a lack of significant difference between those with mild COPD and controls limits its use as a diagnostic aid.

Several studies have explored the potential of DCR to assess pulmonary circulation with changes in pixel contrast and associated signal characteristics during the circulatory cycle being used to generate blood distribution maps [5, 12, 26, 28]. While DCR has been able to demonstrate asymmetric circulation in a number of cases [5], cross-correlation between DCR and perfusion scanning only demonstrated strong correlation in 21% of cases [12], suggesting it may be used as an adjunct to, rather than replacement of, established diagnostic techniques.

While multiple case reports have explored the utility of DCR to demonstrate V/Q mismatch, the lack of large-scale studies comparing it to gold-standard techniques limits its current use [43–46]. Among patients with pulmonary disease, DCR was found to have an accuracy of 62.3% in detecting V/Q mismatch compared to nuclear medicine scanning [26]. Similarly, in patients with lung cancer, DCR-derived ventilation and perfusion measurements have demonstrated moderate correlation with those derived from nuclear medicine scanning; however, this correlation was markedly affected by overlying soft tissue, and reduced in pathological lungs [32].

### Airways imaging

The role of tracheal narrowing in tracheomalacia and excessive dynamic airway collapse (EDAC) is well-recognised [47]. The most commonly utilised diagnostic imaging method is dynamic CT [48] with inspiratory/expiratory slices. DCR, with its ability to assess lung structures throughout the breathing cycle, could theoretically be used to assess tracheal narrowing. Watase et al. [27] have used DCR to identify significant tracheal narrowing during expiration in those with obstructive disease compared to controls. However, measurements were made manually by three observers, without assessment of inter-observer agreement. Similarly, Okhura et al. [25] found tracheal narrowing was significantly higher in those with obstructive disease but failed to demonstrate its utility in diagnosing airflow obstruction.

### Methodological quality

No standardisation exists in the definition of anatomical landmarks used for lung segmentation (for example, inclusion or omission of the left heart border), breathing protocol, or device exposure settings in acquiring DCR images. As is to be expected in a nascent field, few DCR studies have addressed the same hypothesis with the same methodology in the same population. Most DCR studies are small, non-controlled and observational, concerned with the exploration or development of specific radiological techniques related to DCR, and only a handful [22, 24, 31] have recruited larger numbers of subjects, although these too are relatively small. Reproducibility and reliability are therefore difficult to quantify.

## Discussion

This literature review has identified a number of studies exploring the use of DCR in multiple conditions across different populations, highlighting its potential role in clinical practice and demonstrating its ability to capture thoracic motion and the mechanics of pulmonary ventilation and perfusion despite a low radiation dose. However, studies are small and heterogeneous, and few have addressed the same clinical question using the same methodology.

While DCR can demonstrate differences in diaphragm movement between those with respiratory pathology and healthy controls, as well as pre- and post-clinical intervention, the lack of ‘normal’ ranges of diaphragm motion, and the lack of paired, established comparator techniques such as M-mode ultrasound (which can measure diaphragm motion and dysfunction), limits its use in clinical practice. Its use in assessment of other relevant pathologies such as neuromuscular disease remains unexplored. The use of DCR as a screening tool for diaphragm dysfunction appears to be a promising avenue for research.

A shared aim across several studies has been the exploration of the relationship between DCR-calculated lung area or volume and plethysmographic lung volumes, with the implicit goal being to establish the use of DCR as an alternative to PFTs, for example among those whose poor physiological baseline precludes spirometric testing. There is a strong relationship between FVC and lung area, but other clinically useful parameters such FEV_1_ lack a consistent relationship with DCR-derived measurements. These findings likely result from heterogeneity in study design and relatively small sample sizes. The need for specific breathing manoeuvres may prove challenging in some patient cohorts, and studies exploring use of DCR among patients with severe respiratory disease tend to be small in size. There is not enough evidence available for DCR to replace established methods of diagnosis or assessment of lung function in respiratory disease.

Much work has been done to improve the technical ability of DCR to evaluate pulmonary ventilation and perfusion; however its clinical use remains equivocal. Software can map changes in pulmonary blood flow, but automated analysis has not been able to identify perfusion abnormalities reliably, meaning manual (visual) analysis—and the inherent limitations this entails—is still required. This reduces the likelihood that DCR will be able to supplant traditional nuclear medicine techniques in detection of pulmonary or ventilation pathology, at least until improved computation and analysis techniques are developed. While changes in ventilation have been detected between different disease states using DCR, the lack of established ‘normal’ values means further research is required.

There are several barriers to the widespread clinical uptake of DCR. Patients must be able to hold an adequate static position for long enough for image acquisition, which may be challenging for acutely unwell patients. Comparisons of standing and sitting DCR have not yet been performed. Patients with a raised BMI require a higher ionising radiation dose to achieve adequate exposure, and the impact of this on measures such as diaphragmatic excursion and pulmonary perfusion is not known.

Most likely as a result of the novel and investigative nature of the technology, few studies have addressed the same research question in similar populations using similar methods. Likewise, there are no large, multicentre studies amalgamating results or replicating study methods. Studies display significant heterogeneity in their methodology, patient populations, patient positioning, breathing manoeuvres used, and DCR exposure settings and outcomes measured. This, in turn, markedly reduced our ability to numerically synthesise results, limiting the conclusions drawn. It is possible that the heterogeneity of research conducted in DCR research may highlight the technology’s potential strengths. With DCR, analysis of ventilation, perfusion, thoracic structure motion and airways imaging is possible. These data are possible using CT or MRI, but often require a higher-cost and more time- and effort-intensive investigation with the use of contrast dye or inhaled gases. However, no direct comparisons on cost or workflow yet exist to make formal comparisons between DCR and other imaging modalities such as CT, MRI or ultrasound, although work is ongoing [39].

## Conclusions

With the advent of low radiation dose CT systems such as photon counting detector (PCD-) CT [49] and ultra-low-dose thoracic CT [50], DCR requires a clinical niche within dynamic imaging, not reliant solely on its low ionising radiation dose, if it is to be widely utilised. Alongside its ability for straightforward image acquisition and small equipment footprint, the appeal of DCR may lie in its ability to combine diagnostic imaging with physiological motion analysis—a function perhaps best demonstrated in the assessment of diaphragm palsy—alongside ventilation/perfusion information. Given the wealth of information generated from a single DCR series, it may be able to provide a ‘one-stop shop’ to screen for a range of respiratory pathologies—a characteristic that might be of particular use in clinical settings where patients may present with a broad range of differential diagnoses. In such cases, DCR might provide information to allow diagnosis, or at the least act as a rule-out tool prior to more detailed or well-established diagnostic imaging such as CT. However, attaining this goal, along with other exploratory uses for DCR, will require larger trials with comparable methodology to allow for synthesis of results, as well as the inclusion of healthy controls. Such studies may allow DCR to find a unique role in combining traditional static lung imaging and spirometric measures of pulmonary physiology.

## Supplementary Information

**Additional file 1**. Suppementary material.

## Data Availability

Data sharing is not applicable to this article as no datasets were generated or analysed during the current study.

## Abbreviations

COPD: Chronic obstructive pulmonary disease
CT: Computed tomography
DCR: Dynamic chest radiography
ED: Effective Dose
ESD: Entrance surface dose
FEV_1_: Forced expiratory volume of air in 1 s
FOV: Field of view
FPD: Flat panel detector
fps: Frames per second
FVC: Forced vital capacity
GOLD: Global Initiative for Obstructive Lung Disease
ILD: Interstitial lung disease
IQR: Interquartile range
mGy: Milligray
mSv: Millisieverts
PCD: Photon-counting detector
PFT: Pulmonary function tests
PLA: Projected lung area
PLA_exp_: PLA at maximum expiration
PLA_insp_: PLA at full inspiration
PLA_te_: PLA at point of tidal breath out
PLA_ti_: PLA at point of tidal breath in
PPS: Pulmonary perfusion scintigraphy
PRISMA: Preferred Reporting Items for Systematic Reviews and Meta-Analyses
PROSPERO: The International Prospective Register of Systematic Reviews
RSNA: Radiological Society of North America
V/Q: Ventilation/perfusion
ΔPLA: Change in PLA between full inspiration and full expiration
