# Supplementary material for: Dynamic chest radiography: a state-of-the-art review
Source: Insights Imaging. 2023 Jun 19;14:107. doi: 10.1186/s13244-023-01451-4 (PMC10277270; doi:10.1186/s13244-023-01451-4)
Supplement: Supplementary file 1 — Additional file 1. Suppementary material. [file 13244_2023_1451_MOESM1_ESM.pdf]

ELECTRONIC SUPPLEMENTARY MATERIAL

Dynamic Chest Radiography: A State-of-the-Art Review

Supplement 1

Summary of search terms utilised

Search terms
dynamic chest radiography
dynamic chest x-ray
flat panel detector
DCR
DXR
FPD
dynamic chest radiography
dynamic thoracic imaging
dynamic thoracic radiography
functional chest radiography
functional chest x-ray
Image Processing, Computer-Assisted
dynamic
chest x-ray
thoracic

Thorax
X-Rays
x-ray
phrenicography

Supplement 2

Dynamic radiography system equipment and image processing

Dynamic radiography system equipment (Konica Minolta, Inc.)

- CMP200DR 50kW generator (CPI Inc.)
- AeroDR HD 17x17 flat panel detector (Konica Minolta, Inc.)
- Varian Rad-60 Saphire X-ray tube and Optica 60 collimator (Varian Medical Systems Plc.)

Exposure conditions (for both posteroanterior and lateral image series)

- Source to image distance: 200cm
- Focal distance: 180cm
- Tube voltage: 100kV
- Tube current: 80mA*
- Exposure duration of pulsed X-ray: 4ms
- Image capture rate: 6 or 15fps
- Pixel size: 400μm x 400μm
- Matrix size: 1062 x 1062 pixels

- Maximum image area: 42.5cm x 42.5cm
- Processing image density: 16bit
- Tube filter: 1.0mm Al + 0.1mm Cu

** can be increased to 160mA for lateral images*

DCR image analysis workstation: Dell Precision 3620, Intel i7-6700 processor, 16GB RAM, 2TB HDD, Microsoft Windows 10 Pro.

Software: proprietary DCR image analysis package (Konica Minolta, Inc.).

Image format: DICOM

Image processing: automated border detection algorithms define lung area and apex/diaphragm midpoint position. Change in lung area or diaphragm / apex-diaphragm position over time are plotted graphically by software and the points of maximum inspiration/expiration calculated automatically. Position data can be exported for further analysis/processing.

Supplement 3

PRISMA flowchart of study inclusion

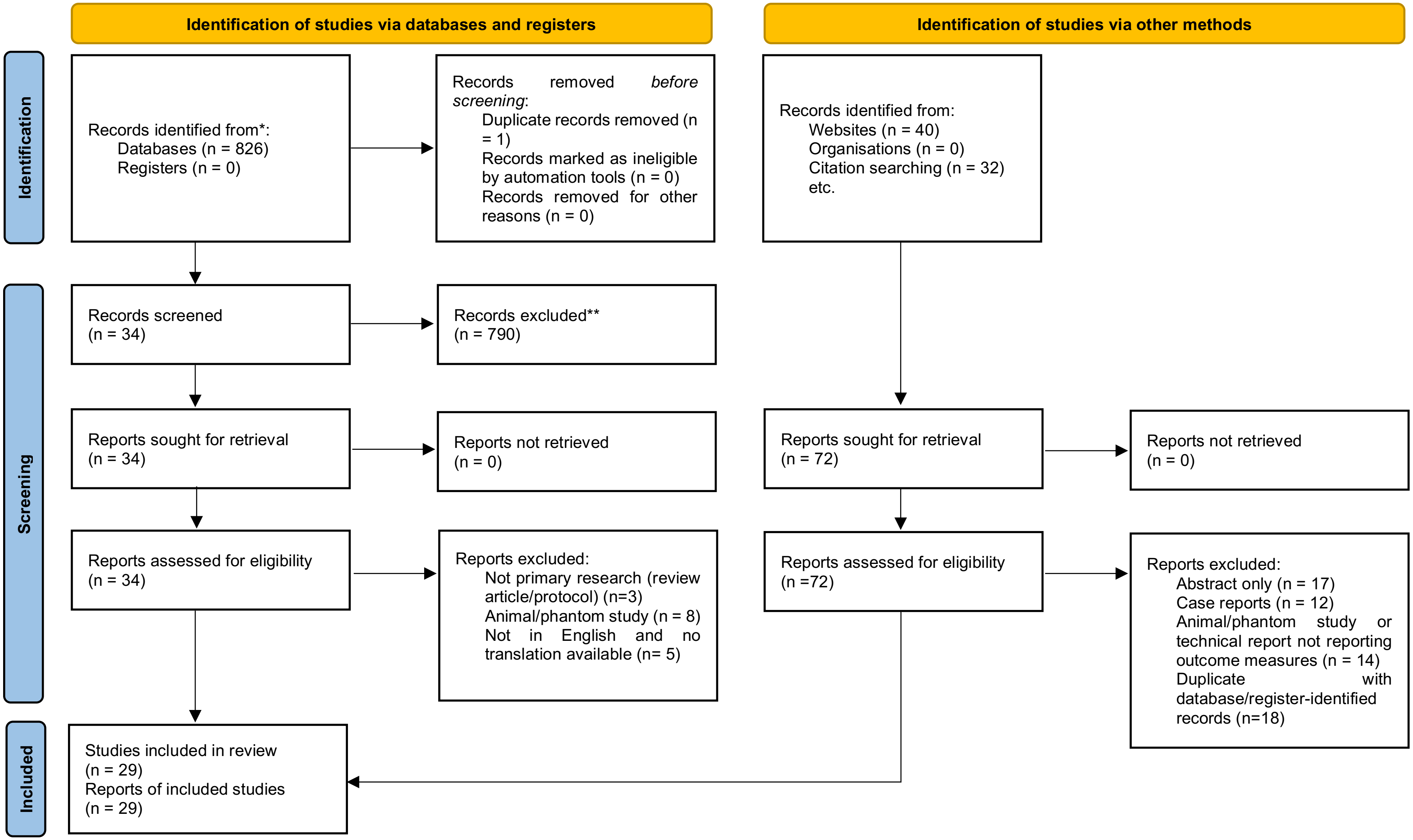

Insights Imaging (2023) Fyles F, Fitzmaurice TS, Robinson RE, Bedi R, Burhan H, Walshaw MJ
